# Association of the Coronavirus Disease 2019 (COVID-19) Outbreak With Enrollment in Cancer Clinical Trials

**DOI:** 10.1001/jamanetworkopen.2020.10651

**Published:** 2020-06-01

**Authors:** Joseph M. Unger, Charles D. Blanke, Michael LeBlanc, Dawn L. Hershman

**Affiliations:** 1 SWOG Cancer Research Network Statistics and Data Management Center, Fred Hutchinson Cancer Research Center, Seattle, Washington; 2 SWOG Cancer Research Network Group Chair’s Office, Oregon Health and Science University Knight Cancer Institute, Portland; 3 Columbia University Medical Center, New York, New York

## Abstract

This cohort study investigates how the coronavirus disease 2019 (COVID-19) pandemic is associated with national enrollment in cancer clinical trials.

## Introduction

Enrollment in clinical trials is key in advancing new treatments for patients with cancer. Poor accrual can result in trials that fail to complete or generate less timely research findings for patients in need of better therapies. The outbreak of coronavirus disease 2019 (COVID-19) has caused severe disruptions in care for many patients—especially patients with cancer, who are more susceptible to infections because of underlying malignant neoplasms or therapy—in an effort to reduce patient and staff exposure and to preserve resources. Given systemwide changes, some institutions in areas with high rates of COVID-19 have stopped all enrollment in interventional trials, while others have made minor changes. An important question is how the COVID-19 pandemic is associated with national enrollment in cancer clinical trials.

## Methods

To address this, we conducted a cohort study to examine initial enrollments in studies conducted by the SWOG Cancer Research Network, a National Cancer Institute–sponsored National Clinical Trials Network group and a member of the National Cancer Institute’s Community Oncology Research Program. Enrollments between January 1 and April 25, 2020, were included. Because trial accrual frequently clusters between Monday and Friday, we aggregated enrollments by week. We followed the Strengthening the Reporting of Observational Studies in Epidemiology (STROBE) reporting guideline for cohort studies. Enrollments were from trials previously approved by an institutional review board; written informed consent was previously obtained for all patients.

Enrollments were compared with the cumulative incidence of confirmed COVID-19 cases in the US.^1^ We examined whether changes in enrollment differed by trial type (treatment vs cancer control and prevention), age (<65 vs ≥65 years), sex, and self-reported race and ethnicity. To explore the potential association of regional outbreak severity with enrollment, we examined patterns according to state-level statistics on cases per 100 000 individuals.^2^ Logistic regression analyses were used to generate odds ratios, with corresponding Wald χ^2^ statistics used to test comparisons. Analyses were conducted in SAS version 9.4 (SAS Institute) and R version 3.4.1 (R Project for Statistical Computing). Statistical significance was set at α = .05, and all tests were 2-tailed.

## Results

Weekly total SWOG enrollments ranged from 125 per week to 150 per week from week 1 (January 1-4) through week 11 (March 8-14), with a mean (SD) enrollment of 137.0 (9.6) (Figure). Beginning in week 12 (March 15-21), enrollment declined to 109; concurrently, the cumulative COVID-19 cases increased from 2918 to 25 697, a nearly 10-fold increase during 7 days. From week 13 (March 22-28) until the end of the study period, weekly accrual did not exceed 74 patients (mean [SD], 66.0 [7.0]), as cumulative COVID-19 cases neared 1 million.

**Figure.**
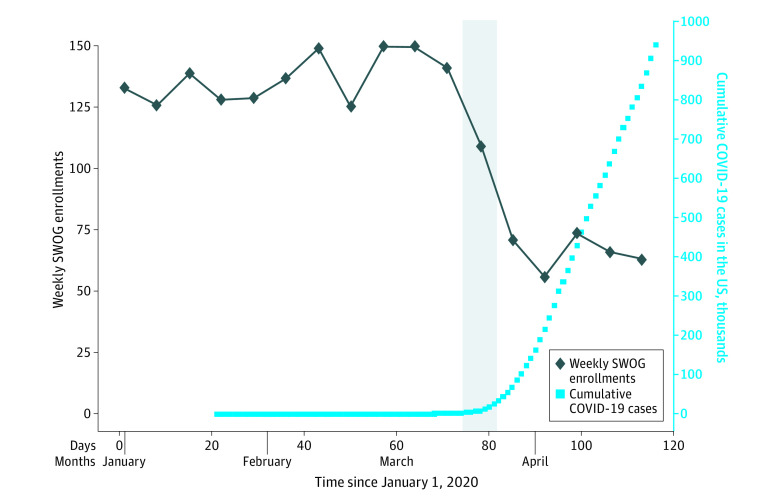
Enrollment to National Cancer Institute–Sponsored Trials vs Coronavirus Disease 2019 (COVID-19) Incidence The first case of COVID-19 in the US was detected on January 21, 2020. For presentation and estimation purposes, the weekly total for the partial week January 1 to 4 was estimated as the mean daily rate for January 2 to 4 multiplied by 7. The vertical gray bar indicates week 12, when COVID-19 cases in the US increased nearly 10-fold.

In total, 1870 patients were enrolled (1431 [76.5%] during weeks 1-11 and 439 [23.5%] during weeks 12-17). There were no significant differences in patterns of decreased enrollment by age, race, or ethnicity (Table). Women patients were marginally less likely to be enrolled in weeks 12 to 17 (odds ratio, 0.77; 95% CI, 0.61-0.99; *P* = .04). Enrollment to cancer control and prevention trials decreased much more than to treatment trials (odds ratio, 0.38; 95% CI, 0.29-0.50; *P* < .001), potentially reflecting an emphasis on offering beneficial treatment to individual patients.^3^ Sites from the states with highest number of COVID-19 cases per 100 000 individuals that composed the upper quintile of enrollments in weeks 1 to 11 were only approximately half as likely to enroll patients in weeks 12 to 17 (odds ratio, 0.56; 95% CI, 0.41-0.76; *P* < .001).

**Table.** Changes in Enrollment by Patient and Trial Characteristics and by Spread of COVID-19

Characteristic	No./total No. (%)			OR (95% CI)^a^	*P* value^b^
	All enrolled patients	Patients enrolled weeks 1-11	Patients enrolled weeks 12-17		
Research setting					
Treatment	1316/1870 (70.4)	948/1431 (66.2)	368/439 (83.8)	1 [Reference]	NA
Cancer control and prevention	554/1870 (29.6)	483/1431 (33.8)	71/439 (16.2)	0.38 (0.29-0.50)	<.001
Age, y					
<65	1272/1870 (68.0)	989/1431 (69.1)	283/439 (64.5)	1 [Reference]	NA
≥65	598/1870 (32.0)	442/1431 (30.9)	156/439 (35.5)	1.23 (0.99-1.55)	.07
Sex^c^					
Men	754/1362 (55.4)	538/1002 (53.7)	216/360 (60.0)	1 [Reference]	NA
Women	608/1362 (44.6)	464/1002 (46.3)	144/360 (40.0)	0.77 (0.61-0.99)	.04
Race					
Other	1563/1794 (86.9)	1186/1372 (86.4)	377/422 (89.3)	1 [Reference]	NA
Black	231/1794 (13.1)	186/1372 (13.6)	45/422 (10.7)	0.76 (0.54-1.08)	.12
Ethnicity					
Non-Hispanic	1699/1822 (93.2)	1307/1400 (93.4)	392/422 (92.9)	1 [Reference]	NA
Hispanic	123/1822 (6.8)	93/1400 (6.6)	30/422 (7.1)	1.08 (0.70-1.65)	.74
State-level COVID-19 case rate per 100 000 residents					
Top quintile^d^	358/1870 (19.1)	301/1431 (21.0)	57/439 (13.0)	0.56 (0.41-0.76)	<.001
Other	1512/1870 (80.9)	1130/1431 (79.0)	382/439 (87.0)	1 [Reference]	NA

Abbreviation: COVID-19, coronavirus disease 2019; OR, odds ratio.

^a^ Shows ORs for being enrolled to a trial from week 12 to 17 (ie, March 15 to April 25) compared with week 1 to 11 (ie, January 1 to March 14); ORs were estimated among patients with known data.

^b^ Based on Wald χ^2^ statistics from logistic regression analyses.

^c^ Among non–sex-specific studies.

^d^ Includes enrollments from sites in New York, New Jersey, Massachusetts, Connecticut, Rhode Island, Louisiana, Michigan, and Delaware.

## Discussion

The COVID-19 pandemic is associated with a notable decrease in enrollment in National Cancer Institute–sponsored cancer clinical trials, especially in areas most affected by COVID-19. The study was limited by its inclusion of enrollment data from only 1 of 4 adult National Cancer Institute network groups; however, the findings are consistent with observed systemwide decreases in accrual.^4^ In response, the National Cancer Institute^6^ and the US Food and Drug Administration^5^ have issued guidance to provide greater flexibility to ensure that patients enrolled in clinical trials are exposed to as little risk as possible during the COVID-19 pandemic. These proactive steps include allowing for remote consent and virtual (ie, telehealth) visits, approaches that may improve the experience of trial participation for patients in the long term. Such measures are vitally important given the critical role government- and industry-sponsored trials play in establishing new oncology treatment options for patients.
